# A putative *Vibrio cholerae* two-component system controls a conserved periplasmic protein in response to the antimicrobial peptide polymyxin B

**DOI:** 10.1371/journal.pone.0186199

**Published:** 2017-10-11

**Authors:** Jyl S. Matson, Jonathan Livny, Victor J. DiRita

**Affiliations:** 1 Department of Medical Microbiology and Immunology, University of Toledo Medical School, Toledo, Ohio, United States of America; 2 Infectious Disease and Microbiome Program, Broad Institute of MIT and Harvard, Cambridge, Massachusetts, United States of America; 3 Department of Microbiology and Molecular Genetics, Michigan State University, East Lansing, Michigan, United States of America; East Carolina University Brody School of Medicine, UNITED STATES

## Abstract

The epidemic pathogen *Vibrio cholerae* senses and responds to different external stresses it encounters in the aquatic environment and in the human host. One stress that *V*. *cholerae* encounters in the host is exposure to antimicrobial peptides on mucosal surfaces. We used massively parallel cDNA sequencing (RNA-Seq) to quantitatively identify the transcriptome of *V*. *cholerae* grown in the presence and absence of sub-lethal concentrations of the antimicrobial peptide polymyxin B. We evaluated the transcriptome of both wild type *V*. *cholerae* and a mutant carrying a deletion of *vc1639*, a putative sensor kinase of an uncharacterized two-component system, under these conditions. In addition to many previously uncharacterized pathways responding with elevated transcript levels to polymyxin B exposure, we confirmed the predicted elevated transcript levels of a previously described LPS modification system in response to polymyxin B exposure. Additionally, we identified the *V*. *cholerae* homologue of *visP* (*ygiW*) as a regulatory target of VC1639. VisP is a conserved periplasmic protein implicated in lipid A modification in *Salmonellae*. This study provides the first systematic analysis of the transcriptional response of *Vibrio cholerae* to polymyxin B, raising important questions for further study regarding mechanisms used by *V*. *cholerae* to sense and respond to envelope stress.

## Introduction

*Vibrio cholerae* is endemic to many regions of the world where it is commonly found in the aquatic environment. When water contaminated with *V*. *cholerae* is ingested by a human, the bacteria colonize the small intestine where they produce cholera toxin, the activity of which causes the profuse watery diarrhea that is the hallmark of cholera disease. In the host, epithelial cells in the crypts of the intestinal lumen (Paneth cells and enterocytes) produce large amounts of antimicrobial peptides, called defensins, that the *V*. *cholerae* and other mucosal pathogens must counter to survive [1]. Defensins, like most antimicrobial peptides, are thought to act by associating with the lipopolysaccharide (LPS) on the bacterial surface (through electrostatic interactions), then permeabilizing the membranes leading to cell death. It is likely that the bacteria also encounter various antimicrobial peptides produced by other organisms in the aquatic environment. Understanding the cellular response by pathogens to host-derived antimicrobial peptides is an active area of research.

The subdivision of *V*. *cholerae* into the classical or El Tor biotype is based on several laboratory tests, one of which is sensitivity to the antimicrobial peptide polymyxin B [2]; classical strains are more sensitive to polymyxin B than El Tor strains. This differential sensitivity is due to a lipid A modification system that adds either a glycine or a diglycine moiety to the lipid A, altering its charge [3]. This system is active in El Tor strains causing them to be relatively resistant to the effect of the antimicrobial peptide. However, a frameshift mutation in one of the genes in the classical biotype renders the system inactive and makes the classical strains more sensitive to polymyxin B [3].

**Fig 1:**
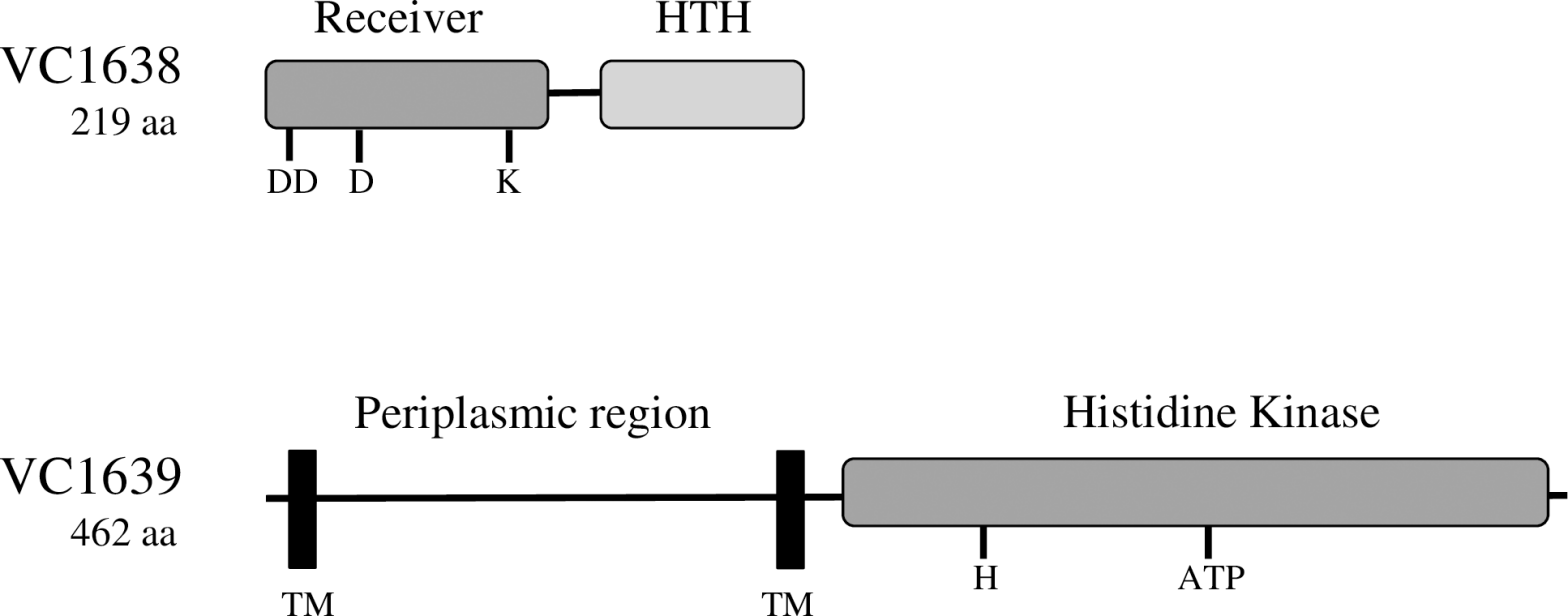
Selected features of the VC1638 and VC1639 proteins. Shaded boxes represent regions sharing sequence similarity to known two-component systems. Selected amino acids conserved among receivers are noted for VC1638: D8, D9, D51, and K101. D51 is predicted to be the phosphorylated aspartate residue. The consensus ATP binding motif (ATP) and the conserved histidine residue (H254) are noted for VC1639. Proposed transmembrane sequences (TM) and the region of VC1639 predicted to be localized to the periplasm are indicated.

Bacterial two-component systems are commonly used to sense changes in the extracellular environment and respond accordingly. They are typically composed of a sensor histidine kinase protein located in the inner membrane and a response regulator protein located in the cytoplasm [4]. The sensor kinase phosphorylates itself in response to a signal and then transfers the phosphate to the response regulator. In many systems, the phosphorylated response regulator goes on to bind DNA and regulate transcription of genes necessary to deal with that particular environmental change. *V*. *cholerae* encodes a variety of two component systems, many of which have not been extensively characterized [5]. We hypothesize that some of these systems play a role in responding to extracellular stresses such as antimicrobial peptide exposure. A genetic screen to identify genes that contribute to polymyxin B resistance identified an insertion in the gene annotated with the locus tag *vc1639*, an uncharacterized putative sensor histidine kinase [6]; a neighboring gene, *vc1638*, encodes a putative response regulator. The domain structure and predicted conserved residues in both proteins resembles the PhoP/PhoQ two-component system in *S*. Typhimurium [7, 8] (Fig 1). This system was implicated as a colonization determinant in an infant mouse model [9], although a defined mutation in *vc1639* does not exhibit a resistance defect in the presence of polymyxin B (unpublished). To more fully understand the role of VC1639 in antimicrobial peptide resistance and disease, we investigated the transcriptome of a strain lacking this two-component system.

Here we compare the transcriptomes of wild type (C6706) and *vc1639* mutant *V*. *cholerae* in the presence and absence of a sublethal concentration of polymyxin B using high throughput sequencing of cDNA libraries (RNA-Seq). We identified a large set of transcripts that were either significantly increased or decreased in abundance upon exposure to polymyxin B, with significant overlap between the two strains. Among the transcripts whose abundance is altered significantly by polymyxin B and by VC1639 is *vca0732*, which encodes a conserved protein VisP (YgiW). VisP in *Salmonella* binds peptidoglycan, possibly through association with NAM and NAG subunits, to modify the activity of a lipid A dioxygenase enzyme LpxO [10]. Unlike *Salmonella* but similar to *E*. *coli*, *V*. *cholerae* does not posses LpxO, raising questions about the role of VisP. Additionally, we observed a polymyxin B- dependent increase in abundance of transcripts for the lipid A modification genes *almEFG*, and their regulators, a two component system encoded by *vc1319/1320*.

## Materials and methods

### Strains and growth conditions

The *V*. *cholerae* El Tor strain C6706 was used in this study. The *Escherichia coli* strains JM101 and DH5αλpir were used for cloning, and SM10λpir was used for conjugation with *V*. *cholerae*. Plasmids used in this study included the suicide vector pKAS32, pTL61T containing a promoterless *lacZ* gene, the arabinose-inducible expression vector pBAD33, and pFlpE, used for FLP-mediated excision of the mariner transposon [11–14]. *E*. *coli* strains were transformed by standard methods [15], and plasmid DNA was introduced into *V*. *cholerae* by electroporation or by filter conjugation with SM10λpir. Antibiotics were used at the following concentrations unless otherwise indicated: polymyxin B, 40 μg/mL; ampicillin, 100 μg/ml; kanamycin, 50 μg/ml; streptomycin, 100 μg/ml; and chloramphenicol, 30 μg/ml (for *E*. *coli*), 5 μg/ml (for *V*. *cholerae*). Expression from pBAD was induced by the addition of L-arabinose to 0.1%.

### Strain and plasmid construction

El Tor (C6706) *V*. *cholerae* containing transposon insertions in *vc1638* and *vc1639* were obtained from a nonredundant transposon insertion library [11]. The majority of the transposon (including a kanamycin resistance cassette and *lacZ* gene) was removed from both strains by FLP-mediated recombination, using the plasmid pFlpE, leaving behind a 192-bp scar, which was validated by PCR and sequencing. Those strains will be referred to as C6706 *vc1638*::*tn* and *vc1639*::*tn*.

The *vca0732* deletion strain was constructed by PCR amplifying 500-bp segments on either side of the open reading frame. The products were joined by SOEing PCR and cloned into suicide vector pKAS32 [14]. The construct was then transformed into *E*. *coli* SM10 (λpir) and moved into *V*. *cholerae* strain C6706 by conjugation.

The *vca0732*::*lacZ* fusion plasmid was constructed by amplifying the upstream 237 nucleotides of *vca0732* (the intergenic region between *vca0731* and *vca0732*) from *V*. *cholerae* C6706 chromosomal DNA using Expand Hi-Fidelity polymerase (Roche Molecular Biochemicals, Indianapolis, IN). After amplification, the PCR products were digested with XhoI and XbaI and ligated into similarly digested pTL61T [13].

Full-length *vc1638* and *vc1639* were amplified from C6706 chromosomal DNA using PCR. After amplification, the PCR products were digested with XbaI and HindIII and ligated into similarly digested pBAD33. All constructs were confirmed by sequencing at the University of Michigan Sequencing Core.

### RNA isolation

RNA was isolated from *V*. *cholerae* grown in LB to mid-log phase and cells were harvested by centrifugation at 4°C. RNA was extracted using TRIzol (Invitrogen, Grand Island, NY) according to the manufacturer’s instructions. Contaminating DNA was removed with at least two treatments of TURBO DNase (Invitrogen). DNase-treated RNA was then cleaned with RNA Clean & Concentrator™-25 columns after each DNase treatment step (Zymo Research, Irvine, CA). Confirmation of DNA removal was assessed using PCR.

### Library construction

Illumina cDNA libraries were constructed in a similar manner to Mandlik et al. [16]. rRNA was depleted from 5 μg of total RNA using the Gram Negative Ribo-Zero™ rRNA Removal Kit (Epicentre, Madison, WI). Removal of contaminating RNA was verified using the Agilent Bioanalyzer RNA 6000 nano chip (Agilent Technologies, Santa Clara, CA). The remaining mRNA was fragmented into 100–500 bp species using the fragmentation buffer from the GeneChip® clean up module kit (Affymetrix, Santa Clara, CA). First-strand cDNA was synthesized using random hexamers, Actinomycin D, and SuperScript III (Life Technologies, Grand Island, NY). Second strand cDNA was synthesized with dUTP replacing dTTP as described by Levin et al. [17]. Double stranded cDNA ends were repaired and adenylated as described in the Illumina Truseq™ RNA sample preparation low throughput (LT) protocol (Illumina, San Diego, CA). Bar-coded Illumina adapters were ligated to the ends of the cDNA libraries and the adapter-cDNA libraries were subsequently treated with Uracil-N-glycosylase (UNG) for 15 min at 37°C, followed by 95°C for 5 min. UNG-treated cDNA was enriched by 8-cycles of PCR using Illumina adapter-specific primers. The final cDNA libraries were cleaned with two treatments of AMPureXP beads (Beckman Coulter, Brea, CA), and sequenced using 50 bp single-end reads on an Illumina HiSeq2000 platform at the University of Michigan Sequencing Core.

### RNA-Seq analysis

Reads were aligned to the *Vibrio cholerae* N16961 reference sequence (RefSeq NC_002505 and NC_002506 for chromosomes I and II, respectively) using BWA [18] version 5.9. Gene annotations were obtained from RefSeq and Rfam [19]. The overall fragment coverage of genomic regions corresponding to features such as ORFs and rRNAs was conducted as described [20]. Differential-expression analysis was conducted with DESeq [21].

### qRT PCR

RNA samples for qRT-PCR were DNase treated, run on an agarose gel to check quality and quantified using a Qubit® 2.0 fluorometer (Life Technologies). Approximately 2.5 μg of each sample was treated with Moloney murine leukemia virus (M-MLV) reverse transcriptase (Invitrogen) according to the manufacturer's specifications. For detection of transcripts, primers amplifying a 200 bp region in the center of the mRNA were used with either SYBR Green Master Mix (Stratagene) on a Stratagene MX3000P thermocycler or FastStart Essential DNA Green Master (Roche) on a LightCycler 96. Primers were designed using the OligoPerfect tool (Invitrogen). Each sample was analyzed in triplicate at least three times, and fold change in expression was calculated using the ΔΔCT method [22], with *recA* transcript levels used as the control.

### β-galactosidase assays

For β-galactosidase assays, *V*. *cholerae* strains were grown overnight at 37°C, then subcultured at a 1:50 dilution into fresh LB and grown for 3 h with aeration. Polymyxin B (40 μg/mL final concentration) was added as indicated and the cultures were grown for an additional hour. Bacteria were then placed on ice and chloramphenicol was added to 0.5 mg/ml. Assays were performed according to the method of Miller [23].

### Survival assays

Antimicrobial peptide susceptibility assays were conducted as described previously [6]. Briefly, overnight cultures of *V*. *cholerae* were subcultured 1:100 into LB and grown for 3 h at 37°C (OD_600_ of ∼0.5). Samples of 5 μl of peptide solution (at a concentration 10 times higher than the final concentration) were placed into wells of a 96-well polystyrene plate, and 45 μl of the bacterial culture was added. After 1 h of incubation at 37°C with shaking, serial dilutions of each culture were plated on LB agar plates. The number of CFU was counted after overnight incubation at 37°C. The percent survival was calculated as follows: survival (%) = (CFU_[peptide treatment]_/CFU_[no treatment]_) × 100.

## Results

### Profiling the transcriptome of *V*. *cholerae* in response to polymyxin B exposure using Illumina-based RNA-Seq

To characterize the *V*. *cholerae* transcriptional response to antimicrobial peptide treatment and the role of the putative VC1639 sensor kinase, we used RNA-Seq analysis to quantitatively identify the transcriptome of *V*. *cholerae* grown in the presence and absence of sublethal concentrations of polymyxin B [6]. Cultures were grown in triplicate to mid-log phase and either treated with 40 μg/mL polymyxin B or left untreated. Cultures were incubated for an additional hour before RNA was isolated. Bar-coded cDNA libraries were generated using methods described by Mandlik et al. [16] and sequenced on the Illumina HiSeq2000 platform at the University of Michigan sequencing core. The average number of reads per sample was 50,269,946 (Table 1), with a mean quality score greater than 35 for every sample representing base calling accuracy of well above 99.9% [24, 25].

**Table 1:** Samples analyzed using RNA-Seq.

Strain/Treatment	Sample #	Number of Reads	Mean quality score
C6706	1	43,465,120	36.22
	2	47,350,615	36.16
	3	41,722,141	36.11
C6706 *vc1639*::*tn*	1	35,207,774	36.70
	2	33,558,048	36.74
	3	32,351,633	36.63
C6706 + polymyxin	1	69,925,737	36.85
	2	60,853,619	36.90
	3	49,536,821	36.82
C6706 *vc1639*::*tn* + polymyxin	1	43,091,866	35.91
	2	91,052,828	35.90
	3	55,123,155	35.86

**Fig 2:**
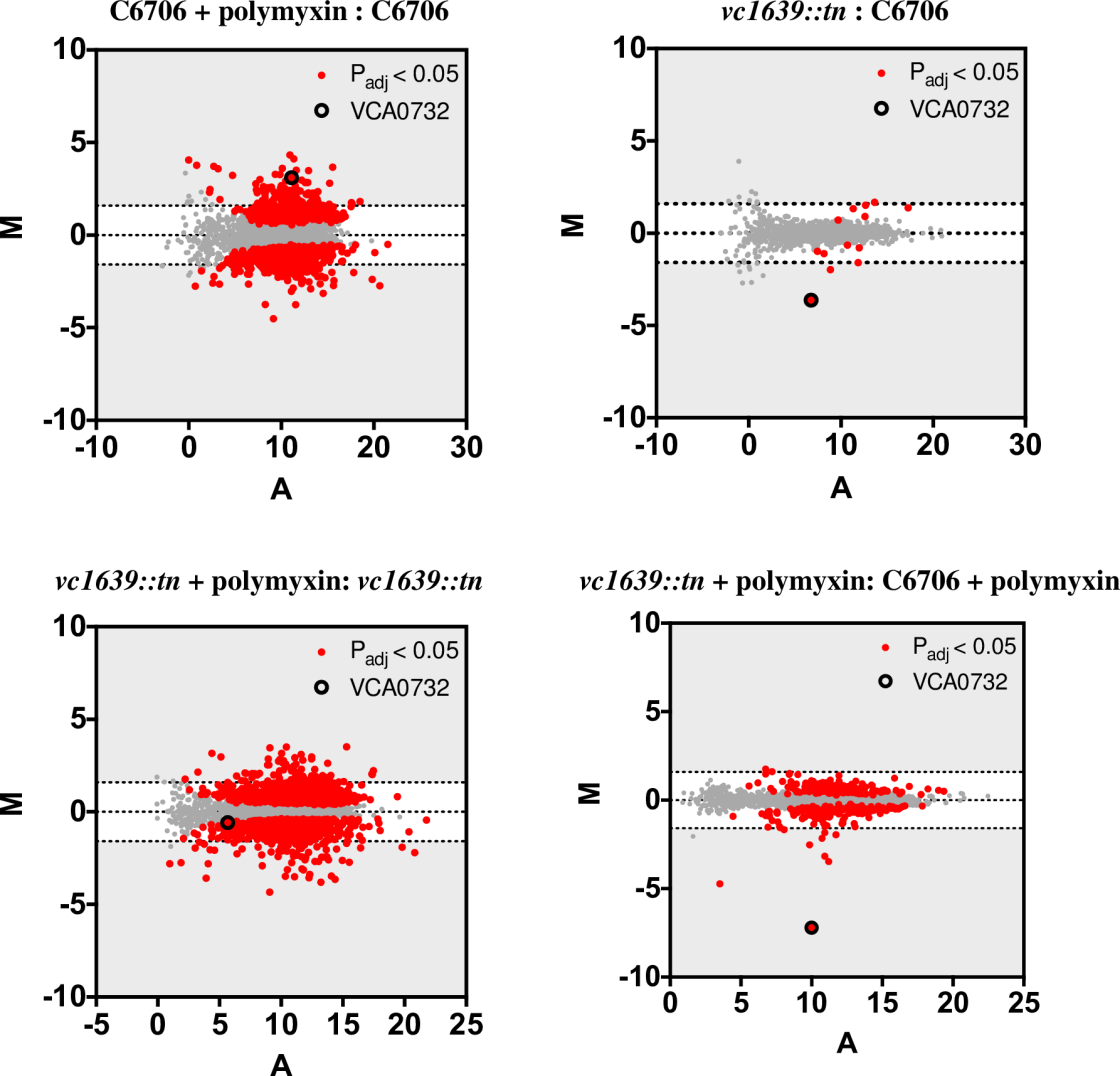
Differential gene expression of *V*. *cholerae* with and without polymyxin B treatment in wild type (C6706) and *vc1639*::*tn* strains. The log_2_ of the ratio of abundances of each gene between the indicated conditions (M) plotted against the average log_2_ of abundance of that gene in all conditions (A). For each plot, (M) and (A) values were generated using DESeq from three biological replicates of each strain and growth condition. Grey regions highlight expression differences of greater- or less-than 3-fold.

We analyzed differential expression of transcripts between the two strains and between polymyxin treated and untreated samples using the variance analysis package DESeq [21]. A number of transcripts showed significantly different abundance (> 3-fold, p_adj_ <0.05) following polymyxin B exposure (Fig 2; indicated in red). Identities of the differentially expressed genes (> 3-fold) between the indicated conditions are listed in S1–S6 Tables. The DESeq analysis of the RNA-Seq data for all annotated *V*. *cholerae* ORFs is listed in S7 Table.

**Fig 3:**
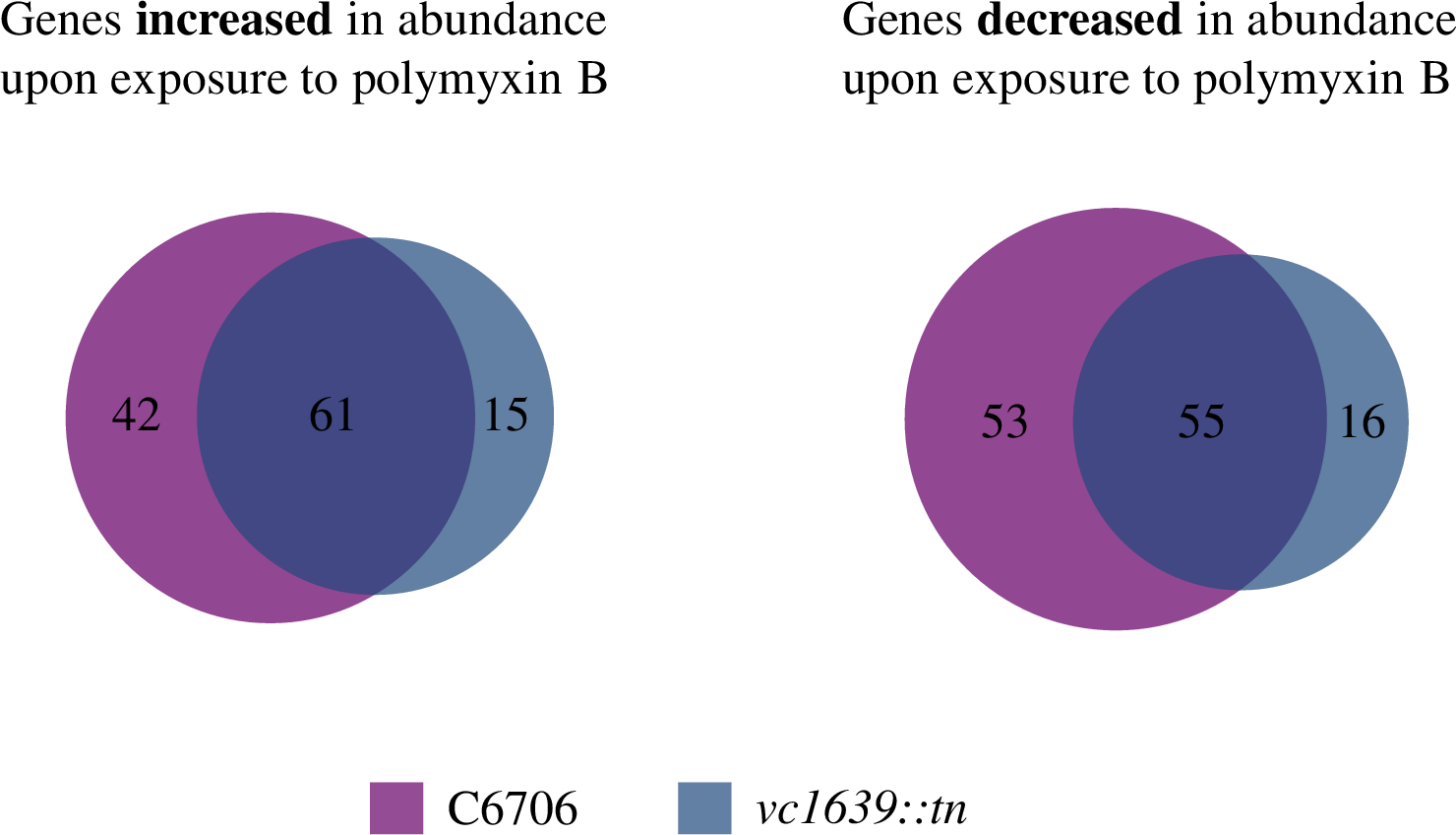
Venn diagram of transcripts with significantly increased or decreased abundance following polymyxin B exposure. The two diagrams show the number of transcripts differentially abundant (> 3-fold, p_adj_ <0.05) following polymyxin B exposure in both the wild type (C6706) and mutant (*vc1639*::*tn*) strain.

There was significant overlap between transcripts differentially abundant following polymyxin exposure in the wild type and *vc1639*::*tn* strain (Fig 3). Transcripts of 103 genes were more highly abundant after polymyxin exposure in wild type *V*. *cholerae* while those of 76 were increased in the *vc1639* mutant strain, with 61 increased transcripts common to both strains. Transcripts of 108 genes showed decreased abundance in response to polymyxin B exposure in our wild type strain and 71 were decreased in the mutant strain, with those of 55 genes commonly decreased in both strains (Fig 3, S1–S4 Tables). Interestingly, among the genes showing increased abundance in both strains, a large number are predicted to encode transporters and outer membrane proteins. However, none of these are homologous to those known to play a role in polymyxin B resistance mechanisms in other bacteria, making it difficult to speculate on their function [26, 27]. We did not identify a large number of transcript levels that were significantly different between the untreated wild type and *vc1639* mutant strain, with only one transcript increased and 14 decreased in the mutant compared to wild type (Table 2).

**Table 2:** Transcripts differentially abundant in the wild type and *vc1639* mutant *V*. *cholerae* strain.

Locus	Gene Name / Function	Log_2_ Fold change	P value
VCA0839	Hypothetical protein	3.688	7.51E-12
VC1027	*moaD*	-2.81029	4.01E-18
VC1243	Hypothetical protein	-5.99164	1.75E-29
VC1333	Hypothetical protein	-4.25442	3.21E-45
VC1374	*dnaK*	-2.68156	4.96E-22
VC1547	Biopolymer transport protein	-1.67947	1.46E-05
VC1639	Sensor histidine kinase	-1.90876	2.38E-11
VCA0246	*ulaA*	-3.40249	4.08E-34
VCA0529	Potassium uptake protein, Kup system	-1.90825	4.85E-07
VCA0707	*uhpC*	-3.40519	5.01E-23
VCA0710	*torT*	-2.30612	1.08E-15
VCA0732	Hypothetical protein	-3.4593	8.12E-31
VCA0744	*glpK*	-1.59124	6.25E-05
VCA0961	Hypothetical protein	-2.55008	3.28E-17
VCA0988	Methyl-accepting chemotaxis protein	-2.00733	7.05E-11

### Elevated transcript abundance from the *almEFG* lipid A modification system and the *vc1319-20* (*carRS*) two-component system after polymyxin B exposure

**Fig 4:**
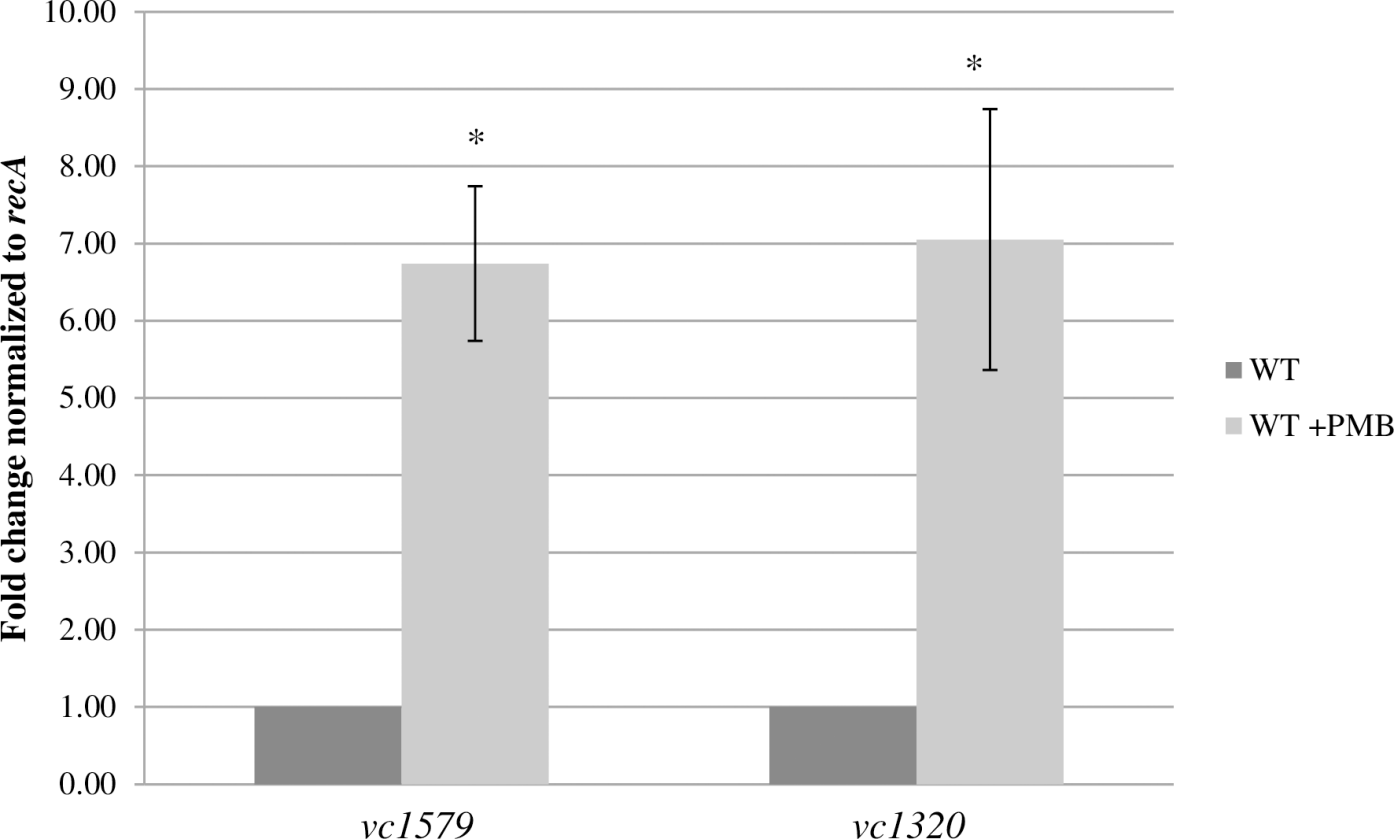
Polymyxin B-induced expression of the *almEFG* lipid A modification system and its associated two component system (*vc1319-20*). Relative expression levels of *vc1579* and *vc1320* were determined using qRT-PCR normalized to *recA* levels. Error bars represent the standard deviation. * *P* < 0.05.

A novel lipid A modification system functions in *V*. *cholerae* to add a glycine or digycline moiety to the lipid A structure [3], resulting in increased resistance to polymyxin B. This system is not functional in classical strains of *V*. *cholerae* due to a frameshift mutation, rendering classical strains more sensitive to the effects of the antimicrobial peptide. This modification system is positively regulated in response to polymyxin B by a two-component system encoded by *vc1319-*20 (*carRS*, *vprAB*) [28, 29]. Consistent with this previous work, our RNA-Seq results indicate that transcripts from the three genes encoding this LPS modification system (*vc1577-79*) are significantly elevated after polymyxin B exposure (Table 3). Additionally, the *vc1319-20* two-component system showed significantly higher transcript levels in the presence of polymyxin. We used qRT-PCR to confirm elevated expression of *vc1579* and *vc1320* transcripts in the presence and absence of polymyxin exposure (Fig 4). These findings suggest that an additional regulatory step(s) upstream of the VC1319-20 two-component system increases levels of these gene products and contributes to lipid A modification in response to antimicrobial peptide exposure.

**Table 3:** Transcriptional response of the lipid A modification system and the *vc1319-20* two-component system in response to polymyxin B exposure as determined by RNA-Seq analysis.

Gene	Log_2_ fold change
VC1577 (*almG*)	2.84
VC1578 (*almF*)	2.74
VC1579 (*almE*)	2.02
VC1319 (*carS*)	2.40
VC1320 (*carR*)	3.18

### Transcript levels from *vca0732* are significantly elevated in response to polymyxin B exposure and dependent on VC1639

One of the goals of this study was to determine the role of the previously uncharacterized VC1638-39 two-component system. As stated previously, we did not identify a large number of genes significantly differentially expressed between wild type and the *vc1639* mutant strain in the absence of polymyxin exposure, and none of these genes is predicted to play an obvious role in *V*. *cholerae* pathogenesis (Table 2). However, our RNA-Seq analysis did provide evidence for regulation of a previously uncharacterized gene, *vca0732*. This gene encodes a predicted periplasmic protein of the bacterial oligonucleotide/oligosaccharide-binding fold (BOF) family [30]. The most closely related gene, *visP* (*ygiW*), is involved in antimicrobial peptide resistance in *S*. Typhimurium [31]. *vca0732* transcript levels are highly abundant in wild type *V*. *cholerae* in response to polymyxin B exposure, and this requires *vc1639* (Tables 2 and 4). We confirmed these results using qRT-PCR for both strains and conditions (Table 4). While the fold change values are not identical between the two methods, the trends are similar.

**Table 4:** *vca0732* transcript levels in response to polymyxin B treatment.

Comparison	RNA-Seq fold change (Log_2_ fold change)	qRT-PCR fold change
*vc1639*::*tn*: C6706	- 12.04 (- 3.46)	- 2.95
C6706 + poly: C6706	+ 8.46 (+ 3.17)	+ 117
*vc1639*::*tn* + poly: *vc1639*::*tn*	- 1.39 (- 0.57)	- 1.43
*vc1639*::*tn* + poly: C6706 + poly	- 118 (- 7.2)	- 265

Fold change values from the RNA-Seq analysis (See also S7 Table) were confirmed by qRT-PCR on independent samples in triplicate normalized to *recA*.

**Fig 5:**
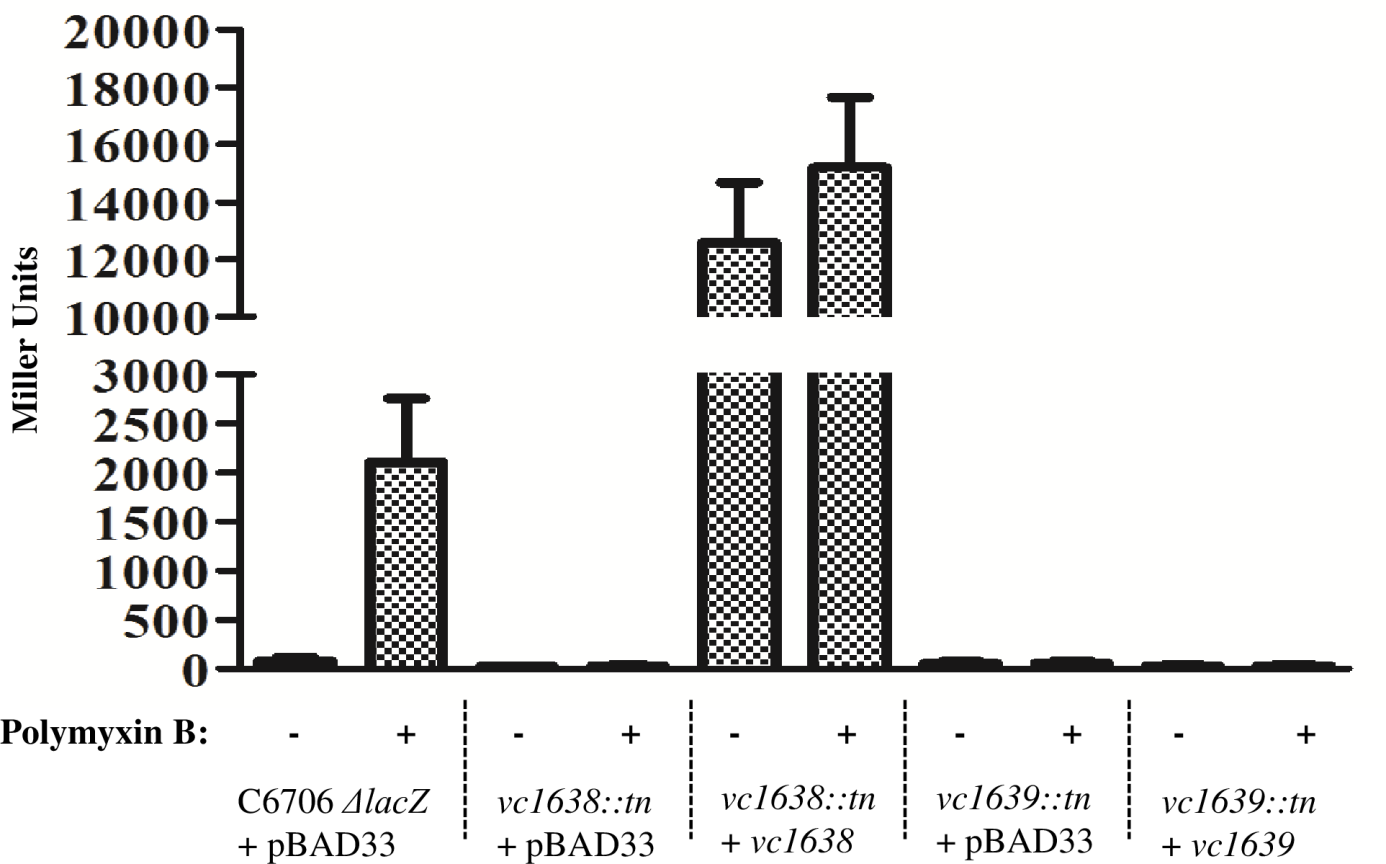
*vca0732* expression is upregulated in response to polymyxin B exposure and dependent on the VC1638-39 two-component system. The promoter region of *vca0732* was cloned in front of a promoterless *lacZ* gene as described in the Materials and Methods. The resulting construct (0732pro) was transformed into strains C6706 *ΔlacZ*, C6706 *vc1638*::*tn*, and C6706 *vc1639*::*tn* (the majority of the transposon, including the *lacZ* gene, was removed by FLP-mediated recombination). pBAD33 was used for complementation studies, with all strains induced by arabinose. Activity of the promoter was assessed in the presence and absence of polymyxin B using the β-galactosidase assay [23]. Error bars represent the standard deviation.

We further confirmed *vca0732* promoter activity using β-galactosidase assays with strains carrying a *vca0732-lacZ* gene fusion. As predicted, *vca0732* promoter activity was elevated upon addition of polymyxin B (Fig 5), and this was dependent on the *vc1638*/*vc1639* putative two-component system. Efforts to individually complement *vc1638* and *vc1639* by expressing the genes in the arabinose-inducible expression vector, pBAD33 gave mixed results. While overexpression of *vc1638* resulted in very high induction of the *vca0732* promoter, irrespective of polymyxin B, two different versions of the *vc1639* construct (differing by the putative initiating methionine codon) did not complement. The reason for the lack of complementation is not clear and additional studies are needed to determine the correct ORF for *vc1639*.

It is unclear whether the two-component system directly regulates *vca0732* expression or if this is an indirect effect. Notably, we did not observe any other genes from the RNA-Seq analysis showing a similar pattern of regulation. VCA0732 does not appear to contribute to antimicrobial peptide resistance in *V*. *cholerae*, as a mutant shows similar survival to that of wild type strains when exposed to polymyxin B, and this is also true of a mutant strain lacking the putative *vca0732* regulator *vc1639* (data not shown). Further work is required to determine the function of VCA0732, as well as to more extensively characterize its regulation by the VC1638-39 two-component system.

## Discussion

RNA-Seq is a powerful tool for providing an unbiased approach to assess transcript abundance under a variety of conditions. It can be applied as to learn more about the function of hypothetical genes. The sequenced genomes of bacteria typically contain numerous genes for which no function can be assigned or no studies have been performed to verify predicted functions. As is the case with most RNA-Seq analysis, several of the differentially regulated genes in our study are annotated as hypotheticals, including *vca0732*. In addition to providing the first systematic analysis of the transcriptional response of *V*. *cholerae* to polymyxin B, the main findings of this study are that i) expression of *vca0732* is strongly induced by exposure to polymyxin B and ii) its expression is dependent on the VC1638-39 two-component system. That no other genes were identified having this same pattern of potential regulation in our study, suggests that a major function of this two-component system is to activate expression of *vca0732*. As yet, the role of this activation and how it might enhance the fitness of *V*. *cholerae* is not clear. *vca0732* is predicted to encode a periplasmic bacterial OB-fold protein, which is a group of currently unknown function [30]. The most closely related protein to VCA0732 is VisP (YgiW) of *Salmonella enterica* serovar Typhimurium. A mutation in *visP* causes *S*. Typhimurium to become moderately sensitive to polymyxin B [10], whereas a mutation in *vca0732* in *V*. *cholerae* did not increase polymyxin sensitivity (data not shown). One of the functions of VisP in *S*. Typhimurium is to inhibit the lipid A modifying activity of LpxO, leading to increased resistance to stress within macrophage vacuoles and promoting systemic disease [10]. Several bacterial pathogens including *V*. *cholerae* and *E*. *coli* have a VisP homologue but no LpxO, making the function of these protein more difficult to determine. Further work is necessary to identify LpxO-independent activities of these VisP homologues.

In *S*. Typhimurium, expression of *visP* is regulated by an adjacent two-component system, QseBC (PreAB) [32]. The QseBC two-component system regulates its own expression in addition to *visP* and several other virulence genes. While QseBC and VC1638-39 are both two-component systems (with 20% identity by clustalW alignment; S1 Fig) that regulate similar gene products (*vca0732* and *visP*) there are several features that distinguish them in other ways. First, *vc1638-39* are not located adjacently to *vca0732*, even residing on different chromosomes. Second, our RNA-Seq data do not reveal any evidence that VC1638-39 regulates another two-component system homologous to PmrAB, as is the case for QseBC in *S*. Typhimurium. Third, VC1638-39 have no verified role in the pathogenesis of *V*. *cholerae*, as has been shown for QseBC in *Salmonella*. Finally, we observed no sensitivity to polymyxin B for mutants in either the *vc1638-39* two-component system or in *vca0732*, despite the fact that *vca0732* is so highly upregulated by exposure to the antimicrobial peptide (data not shown). Further work is required to characterize the function of this hypothetical gene, its role in responding to polymyxin exposure, and its regulation by the VC1638-39 two-component system.

Recent work from the Raivio laboratory aimed to further characterize the Cpx regulon and its role in the envelope stress response in *V*. *cholerae*. Microarray analysis of a strain grown in virulence-inducing conditions and overexpressing CpxR was used to identify genes showing differential expression in this background. Of note, both *vc1639* and *vca0732* showed significantly increased expression [33]. This is additional evidence suggesting that VCA0732 and the VC1638-39 two-component system are connected to stress response pathways in *V*. *cholerae*. Further work is needed to verify the microarray data and to determine whether CpxR regulation of these genes is direct or indirect.

Our RNA-Seq analysis demonstrated elevated levels of transcripts encoding *almEFG* lipid A modification system upon exposure to polymyxin B, and that this transcription is also controlled by the VC1319-20 two-component regulatory system. This verifies recent previous work [28, 29] and demonstrates that this modification system is likely not constitutively expressed but rather is specifically regulated by environmental factors. We determined that the transcripts from the two-component system are elevated in response to polymyxin exposure, indicating that additional regulatory steps exist upstream of VC1319-20. This two-component system–termed CarRS–was originally identified as regulating expression of genes in response to calcium concentration [34]. As a part of that study, the authors performed microarray analysis on *carR* and *carS* deletion strains. Genes with altered expression levels in the absence of the two-component system included those encoding the lipid A modification system (*vc1577-79*), which were all significantly downregulated in the absence of either regulator and were also affected by the concentration of calcium in the environment [34]. That work demonstrated that this two-component system is a negative regulator of *vps* expression and thus biofilm formation. The *almEFG* lipid A modification system also plays a role in intestinal colonization (12). A mutation in *almE* led to reduced colonization of the infant mouse intestine as compared to wild type *V*. *cholerae*. The absence of the VC1319-20 two-component system also results in a colonization defect, albeit a smaller one (12). Based on the two studies regarding the two-component system, the observed defect in colonization could be due to lower expression of the lipid A modification system or due to elevated biofilm production, or some combination of both phenotypes. Further work on the VC1319-20 two-component system and its regulation are required to determine the cause of the observed defect.

RNA-Seq has emerged as a very powerful approach for mapping transcriptomes and analyzing gene expression in a variety of organisms. This technique is especially powerful in bacteria as their small genome size allows for incredibly high coverage and low cost due to the ability to multiplex. While this is not the first report using RNA-Seq to analyze gene expression in *V*. *cholerae*, this analysis adds to previous studies in that RNA-Seq data can be compared across experiments unlike other methodologies, giving additional information that could benefit many other researchers working on different questions in the same organism. These data will be even more informative in the future if similar studies are performed to investigate the transcriptomes of *V*. *cholerae* in response to other stressors. By comparing those types of studies, we will be able to determine global, non-specific responses to extracellular stress and elucidate which pathways are specific to a particular insult.

## Supporting information

S1 Table Genes increased in abundance in C6706 treated with polymyxin compared to untreated C6706. (XLSX)

S2 Table Genes decreased in abundance in C6706 treated with polymyxin compared to untreated C6706. (XLSX)

S3 Table Genes increased in abundance in Δ*vc1639* treated with polymyxin compared to untreated Δ*vc1639*. (XLSX)

S4 Table Genes decreased in abundance in Δ*vc1639* treated with polymyxin compared to untreated Δ*vc1639*. (XLSX)

S5 Table Genes increased in abundance in Δ*vc1639* treated with polymyxin compared to C6706 treated with polymyxin. (XLSX)

S6 Table Genes decreased in abundance in Δ*vc1639* treated with polymyxin compared to C6706 treated with polymyxin. (XLSX)

S7 Table Results of DEseq analysis of RNA-Seq data for all annotated *V*. *cholerae* ORFs. padj is the adusted P values for each gene and resvarA/B are the variance values within each set of biological replicates.
(XLSX)

S1 Fig ClustalW alignment of VC1639 from *V*. *cholerae* and QseC from *S*. Typhimurium. (PDF)
