# Supplementary material for: A putative Vibrio cholerae two-component system controls a conserved periplasmic protein in response to the antimicrobial peptide polymyxin B
Source: PLoS One. 2017 Oct 11;12(10):e0186199. doi: 10.1371/journal.pone.0186199 (PMC5636140; doi:10.1371/journal.pone.0186199)
Supplement: S1 Fig — (PDF) [file pone.0186199.s008.pdf]

```
gi|21759377|sp|Q8Z3P2.1|QSEC_S      ---------MKLTQRLSLRVRLTLIFLILVSITWAISSFVAWRKTTDNVD 41
gi|15641644|ref|NP_231276.1|        MGISCMPIKSRALRHLSLKTRLLLAAALWLGAMILAAGYLIPNLIHQYLL 50
                                              :  ::***:.** *   : :.     :.::  .   : :

gi|21759377|sp|Q8Z3P2.1|QSEC_S      ELFDTQLMLFARRLSTLDLNELNAPQRMAHTPKKLKHGHIDDDALAFAIF 91
gi|15641644|ref|NP_231276.1|        EDVQNQLQLS---MDEITANLEANPQGQLTLTTRLADPRFTQPYSGLYWS 97
                                    * .:.** *    :. :  *    **     ..:* . :: :   .:

gi|21759377|sp|Q8Z3P2.1|QSEC_S      SADGKMLLHDGDNGQDIPYRYRREGFDNGYLKDDND----LWRFLWLNSA 137
gi|15641644|ref|NP_231276.1|        ATLGDQTLRS-RSLWDRFLTEETHVHGTIYLGAKEESLIVLKRTVYLPEF 146
                                    :: *.  *:.  .  *     . . ... **  .::    * * ::* .

gi|21759377|sp|Q8Z3P2.1|QSEC_S      DGKYRIVVGQEWDYREDMALAIVAAQLTPWLIALPFMLLILLLLLHRELR 187
gi|15641644|ref|NP_231276.1|        SQPITITIGLDDAPLKATLQRVSKELWMILALLFSGILVLTGVQVTWSLR 196
                                    .    *.:* :    :     :         : :. :*::  : :  .**

gi|21759377|sp|Q8Z3P2.1|QSEC_S      PLKKLAQALRFRSPESETPLDAKGVPSEVRPLVEALNQLFSRIHSMMVRE 237
gi|15641644|ref|NP_231276.1|        PLSKMQRELSALRDGQQNALNGD-YPQEISPLVNDLNALLFHYQELLERA 245
                                    **.*: : *      .:..*:..  *.*: ***: ** *: : :.:: *

gi|21759377|sp|Q8Z3P2.1|QSEC_S      RRFTSDAAHELRSPLAALKVQTEVAQLSGDDPLSRDKALTQLHAGIDRAT 287
gi|15641644|ref|NP_231276.1|        RHHAGNLSHALKTPLSVLKN--EVTQLN------DAQLRIQLQPSVEQIQ 287
                                    *:.:.: :* *::**:.**   **:**.        :   **:..:::

gi|21759377|sp|Q8Z3P2.1|QSEC_S      RLVDQLLTLSRLDSLNNLQDVAEISLEELLQSAVMDIYHPAQQANIDVRL 337
gi|15641644|ref|NP_231276.1|        QQIDYHLGRARMAGSANILSVKTAPSAR--VDAISQAFDKVYAERGITLV 335
                                    : :*  *  :*: .  *: .*   .  .   .*: : :. .   .  . :

gi|21759377|sp|Q8Z3P2.1|QSEC_S      QLNAHDVIRTGQPLLLSLLVRNLLDNAIRYSPQGSVVDVTLHAR---SFT 384
gi|15641644|ref|NP_231276.1|        NELDSDLEVAVEPTDLDEMIGNLLENSYKWANSLIRVHSQILSDDWVQIC 385
                                    :    *:  : :*  *. :: ***:*: ::: .   *.  : :    .:

gi|21759377|sp|Q8Z3P2.1|QSEC_S      VRDNGPGVAPEILTHIGERFYRPPGQSVTGSGLGLSIVRRIATLHGMTVS 434
gi|15641644|ref|NP_231276.1|        VEDDGAGISATNREKALKRGVR-LDETTPGTGLGLNIVSEMAHSYRGALA 434
                                    *.*:*.*::.    :  :*  *  .::..*:****.** .:*  :  :::

gi|21759377|sp|Q8Z3P2.1|QSEC_S      FGNAAEGGFEAVVRW------------- 449
gi|15641644|ref|NP_231276.1|        LGESQLGGLKATLTLKQPTRAAKAVKSH 462
                                    :*::  **::*.:
```

Figure S1. ClustalW alignment of VC1639 from *V. cholerae* and QseC from *S.* Typhimurium.
